# Pseudorabies virus-induced expression and antiviral activity of type I or type III interferon depend on the type of infected epithelial cell

**DOI:** 10.3389/fimmu.2022.1016982

**Published:** 2022-11-02

**Authors:** Yue Yin, Jinglin Ma, Cliff Van Waesberghe, Bert Devriendt, Herman W. Favoreel

**Affiliations:** Department of Translational Physiology, Infectiology and Public Health, Faculty of Veterinary Medicine, Ghent University, Merelbeke, Belgium

**Keywords:** epithelial cell, pseudorabies virus, alphaherpesvirus, type I interferon, type III interferon, respiratory, gastrointestinal, kidney

## Abstract

Type I and III Interferons (IFNs) are the initial antiviral cytokines produced in response to virus infection. These IFNs in turn bind to their respective receptors, trigger JAK-STAT signaling and induce the expression of IFN-stimulated genes (ISGs) to engage antiviral functions. Unlike the receptor for type I IFNs, which is broadly expressed, the expression of the type III IFN receptor is mainly confined to epithelial cells that line mucosal surfaces. Accumulating evidence has shown that type III IFNs may play a unique role in protecting mucosal surfaces against viral challenges. The porcine alphaherpesvirus pseudorabies virus (PRV) causes huge economic losses to the pig industry worldwide. PRV first replicates in the respiratory tract, followed by spread *via* neurons and *via* lymph and blood vessels to the central nervous system and internal organs, e.g. the kidney, lungs and intestinal tract. In this study, we investigate whether PRV triggers the expression of type I and III IFNs and whether these IFNs exert antiviral activity against PRV in different porcine epithelial cells: porcine kidney epithelial cells (PK-15), primary respiratory epithelial cells (PoREC) and intestinal porcine epithelial cells (IPEC-J2). We show that PRV triggers a multiplicity of infection-dependent type I IFN response and a prominent III IFN response in PK-15 cells, a multiplicity of infection-dependent expression of both types of IFN in IPEC-J2 cells and virtually no expression of either IFN in PoREC. Pretreatment of the different cell types with equal amounts of porcine IFN-λ3 (type III IFN) or porcine IFN-α (type I IFN) showed that IFN-α, but not IFN-λ3, suppressed PRV replication and spread in PK-15 cells, whereas the opposite was observed in IPEC-J2 cells and both types of IFN showed anti-PRV activity in PoREC cells, although the antiviral activity of IFN-α was more potent than that of IFN-λ3 in the latter cell type. In conclusion, the current data show that PRV-induced type I and III IFN responses and their antiviral activity depend to a large extent on the epithelial cell type used, and for the first time show that type III IFN displays antiviral activity against PRV in epithelial cells from the respiratory and particularly the intestinal tract.

## Introduction

Mucosal surfaces, including the gastrointestinal tract, respiratory tract and reproductive tract are mainly composed of epithelial cells. They provide a crucial physical barrier between the body and the external environment and present the initial target cells for the establishment of viral infections (1, 2). Meanwhile they also have evolved unique defense mechanisms to protect against virus infection.

Type I and/or type III interferons (IFNs) are the initial antiviral cytokines that are produced as part of the innate immune response against virus infection, before activation of the adaptive immune system. Interferons are typically produced upon recognition of pathogen-associated molecular patterns (PAMPs) by cytosolic (e.g., RIG-I, MDA5, and cGAS) or endosomal (e.g., TLR3 and TLR9) pattern recognition receptors (PRRs). During virus infections, viral nucleic acids, i.e. viral DNA and/or RNA, are the major PAMPs. The activation of PRRs leads to the activation of several transcription factors, including interferon regulatory factor 3/7 (IRF3, IRF7) and nuclear factor-κB (NF-κB) to induce transcription, translation, and secretion of type I and/or type III IFNs and pro-inflammatory cytokines (3, 4). Type I and III IFNs bind to their respective receptors which, in both cases, results in activation of the JAK-STAT signaling pathway, which culminates in the expression of a plethora of IFN-stimulated genes (ISGs) that restrict viral replication and spread. Type I IFNs such as IFN-α and IFN-β bind the two chains of the type I IFN receptor (IFNAR1/2), whereas type III IFNs bind the two chains of the type III IFN receptor (IL-10R2, IFNLR1). Whereas the type I IFN receptor is expressed broadly, the expression of the IFNLR1 receptor is mainly restricted to mucosal epithelial cells. Accumulating evidence has shown that type III IFNs are of particular importance in protecting mucosal surfaces against viral challenges (5, 6).

Pseudorabies virus (PRV) (also referred to as suid herpesvirus 1 or Aujeszky’s disease virus) belongs to the subfamily *Alphaherpesvirinae* of the herpesviruses and is an enveloped, double-stranded DNA virus. PRV has a very broad host range, including most mammals and some avian species (7). Pigs are the only natural host and reservoir of PRV. PRV first replicates in the respiratory tract, followed by spread *via* neurons and *via* lymph and blood vessels to the central nervous system and internal organs, e.g. the kidney, lungs, and intestinal tract (8). PRV causes Aujeszky’s disease or pseudorabies (PR) in pigs, with high mortality in young piglets and reduced growth and reproductive failure in older pigs. Some of the major clinical symptoms include respiratory problems, diarrhea, vomiting, and nervous system disorders. The consequences of PRV infection result in huge economic losses to the pig industry worldwide (9).

Although type I IFNs have been shown to display antiviral activity against several alphaherpesviruses, the antiviral activity of type III IFNs has thus far been shown against the human alphaherpesvirus herpes simplex virus type 1 and 2 (HSV-1 and HSV-2) in human corneal explants and vaginal mucosa, respectively (10, 11). With regard to PRV, antiviral activity has been reported using recombinant porcine IFN-λ1 in PK-15 cells using the wild type Ea strain and using porcine IFN-λ3 in PK-15 cells using an attenuated gEnull/gInull PRV strain (12, 13). There is currently no information available about whether type III IFN suppress alphaherpesviruses infection in cells of the respiratory epithelium or intestinal epithelium or whether porcine IFN-λ3 displays antiviral activity against a wild type PRV strain. PRV is widely used as a model system to study general aspects of alphaherpesvirus biology. In the current study, we investigate the type I and III IFN response to PRV infection and the antiviral activity of both types of IFN in porcine kidney epithelial cells (PK-15), primary respiratory epithelial cells (PoREC) and intestinal porcine epithelial cells (IPEC-J2). We show that type I and III IFNs are produced in response to PRV infection in PK-15 cells and IPEC-J2 cells, with limited to no detection of either type of IFN in PoREC. Pretreatment of cells with the same amount of porcine IFN-λ3 (type III IFN) or porcine IFN-α (type I IFN) indicated that, depending on the cell type, both types of IFN may display antiviral activity (PoREC) or either one of the IFN types may display antiviral activity (IFN-λ3 in IPEC-J2 cells and IFN-α in PK-15 cells).

## Material and methods

### Cell cultures, virus and interferon

Porcine kidney-15 (PK-15) cells were cultured at 37°C with 5% CO2, in minimal essential medium (MEM; ThermoFisher), supplemented with 10% inactivated fetal bovine serum (FBS), 100-U/ml penicillin, 0.1-mg/ml streptomycin, 50-ug/ml gentamicin.

The intestinal porcine epithelial cell line J2 (IPEC-J2) was cultured at 37°C with 5% CO2 in Dulbecco’s Modified Eagle’s Medium Nutrient Mixture F-12 (DMEM/F12, Gibco) supplemented with 0.1 mM HEPES (Gibco, USA), 10% inactivated fetal bovine serum (FBS), 100 U/ml penicillin, 0.1 mg/ml streptomycin, 1% insulin-transferrin-selenium (ITS), 2% l-glutamine, and 5 ng/ml epidermal growth factor (EGF).

PRV strain Kaplan was described before and was kindly provided to us by T. C. Mettenleiter (Friedrich-Loeffler-Institute, Germany) (14).

Porcine recombinant IFN-α and IFN-λ3 were purchased from Kingfisher Biotech (USA).

### Isolation of primary PoRECs

Isolation and culture of porcine primary respiratory epithelial cells (PoREC) was performed as previously described (15). In brief, tracheae were collected from healthy 7-weeks-old piglets and were immediately placed in ice-cold transport solution (phosphate buffered saline (PBS), supplemented with 100U/mL penicillin, 0.1mg/mL streptomycin, 0.1μg/mL gentamycin, 0.1mg/ml kanamycin [Sigma-Aldrich] and 250ng/mL fungizone (Gibco, Thermo Fisher Scientific)) for transportation to the laboratory. Connective tissue and fat were removed from the trachea, which were then washed with PBS to remove red blood cells and incubated in a Pronase/DNase I solutiuon for 72 h at 4°C. After incubation, cells were harvested and then added to uncoated plastic petri dish (Nunc; Thermo Fisher Scientific) for 2h to remove fibroblasts by plastic adherence. Epithelial cells were seeded at a concentration of 1.2 million cells/well in 12-well plate transwells with a 0.4-μm pore size (catalog number 3460; Costar; Corning) that were pre-coated with type IV collagen (catalog number C5533; Sigma-Aldrich). Cells were cultivated in Afi1 medium (DMEM plus Ham’s F-12 medium, supplemented with 5% FBS, 1% MEM nonessential amino acids (Gibco, Thermo Fisher Scientific), 100U/ml penicillin, 0.1mg/ml streptomycin, 1.25 μg/ml amphotericin B). One day after seeding, cells were carefully washed with DMEM/F12 medium and Afi2 medium supplemented with 2% Ultroser G was added to the bottom of the transwell, resulting in an air-liquid interface to simulate conditions of the respiratory tract. PoREC were incubated at 37°C with 5% CO2 until they reached full confluence, upon which they were apically infected with PRV Kaplan at multiplicity of infection (MOI) of 10 for 24h.

### Cell viability assay

Confluent PK-15, PoREC, IPEC-J2 cells were treated with 300ng/ml IFN-α or IFN-λ3 for 24h, incubated for 20 min at 37°C with accutase and collected in 96-well V-bottom plates. Cells were washed two times in PBS between each step. Cells were fixed with Fix buffer (BD Biosciences) for 20min at 4°C as positive control for propidium iodide (PI) staining, which was used to measure cell viability. Flow cytometry was performed using a NovoCyte Flow Cytometer (ACEA Biosciences, Agilent, Santa Carla, CA, USA), and samples were analyzed with NovoExpress software (ACEA Biosciences).

### RNA isolation and RT-QPCR

RNA isolation and RT-QPCR were performed as described before (16).The primers used in this assay are listed in **Table 1**.

**Table 1:** Forward and reverse primer sequences used in real-time PCR assays.

Target, (GenBank Accession No.)	Forward primer 5’-3’	Reverse primer 5’-3’
28S (17)	GGGCCGAAACGATCTCAACC	GCCGGGCTTCTTACCCAT
pIFN-λ1 (18)	GGTGCTGGCGACTGTGATG	GATTGGAACTGGCCCATGTG
pIFN-λ3 (19)	ACTTGGCCCAGTTCAAGTCT	CATCCTTGGCCCTCTTGA
pIFN-α (20)	TCTCATGCACCAGAGCCA	CCTGGACCACAGAAGGGA
OAS1(NM_002534.4)	GAGCTGCAGCGAGACTTCCT	TGCTTGACAAGGCGGATGA
ISG15(NM_001128469.3)	AGCACAGTCCTGTTGATGGTG	CAGAACTGGTCAGCTTGCACG
ISG54(XM_005671264.3)	GCCCTAAGGACCCAGAAGTCA	CGAGGAGGTGGCCAGTTATC
IFIT3 (21)	GCACCAAATTCATGGTATCTCC	TTCCTTCCTGTCTCTGTCAGCC

### SDS-PAGE and Western blot analysis

SDS-PAGE and Western blot analysis were performed as described before (16). Rabbit anti-IFNAR2 polyclonal antibody (catalog no. GTX105770; GeneTex)(1:500 dilution), rabbit anti-IFNLR1 polyclonal antibody (catalog no. ARP48070_P050; Aviva Systems Biology)(1:500 dilution) and goat anti-rabbit IgG (catalog number P0448; Dako)(1:3,000 dilution) were used.

### Titrations

PK-15, PoREC and IPEC-J2 were either left untreated or pre-treated with the indicated concentrations of IFN for 24 h. Cells were then inoculated with PRV strain Kaplan at an MOI of 0.1. At 2h post inoculation (hpi), cells were washed with PBS and incubated with sodium citrate buffer (pH 3.0; 40 mM sodium citrate, 10 mM KCl, and 135 mM NaCl) for 2 min at room temperature to inactivate residual virus, followed by several washing steps before adding fresh medium. At 24hpi, PRV-infected cell supernatants were harvested and immediately frozen at −80°C. Extracellular titers were determined on ST cells. To this end, ST cells were seeded in 96-well-plates and inoculated with serial 10-fold dilutions of PRV-infected cell supernatants and, after 7 days, extracellular virus titer was calculated in plaque forming units (PFU) per milliliter (PFU/ml).

### Plaque assays

PK-15, PoREC and IPEC-J2 cells were either left untreated or pretreated with the indicated concentrations of IFN for 24 h, then infected with PRV at 1,000 PFU/well. At 2hpi, medium was replaced by 1mL of semisolid medium consisting of a 1:1 mixture of 2 × DMEM with 4% FBS and 2% methylcellulose solution. At 24hpi, cells were fixed with 3% paraformaldehyde at room temperature for 15 min followed by three washing steps with PBS and subsequent permeabilization using 0.1% Triton X-100 at room temperature for 2 min. Afterwards, cells were washed three times with PBS. For PoREC cells, cells were incubated with antibodies against the epithelial cell-specific marker cytokeratin (rabbit polyclonal anti-pan-cytokeratin antibody (1:200); Abcam) overnight at 4°C and fluorochrome-linked goat anti-rabbit IgG secondary antibodies (1:200; Invitrogen) for 1 h at 37°C. For PRV plaque detection, cells were incubated in fluorescein isothiocyanate (FITC)-labeled porcine polyclonal anti-PRV antibodies (1:200) at 37°C for 1h, diluted in incubation buffer (10% FBS diluted in PBS). After three washing steps with PBS, Hoechst 33342 (1:200; Invitrogen) diluted in PBS was added at room temperature for 10 min to counterstain the cell nuclei. After three washing steps, coverslips were mounted on microscope slides in glycerin-DABCO. Samples were imaged using a Leica SPE confocal microscope (Leica) and were analyze with Image J software.

### Statistical analysis

Statistical analyses were performed using Prism 7 (GraphPad Software). Statistical differences among the experimental groups were determined by unpaired t tests.

## Results

### In PK-15 cells, PRV triggers a multiplicity of infection (MOI)-dependent type I and prominent type III IFN response and IFN-α displays anti-PRV activity

Most types of cells are able to produce type I interferons upon virus infection. However, type III interferons are typically only produced in epithelial cells and specialized interferon-producing innate immune cells, the plasmacytoid dendritic cells (pDC) (22). In combination with the fact that the expression of the type III IFN receptor is largely restricted to epithelial cells, type III IFNs particularly provide a local antiviral response in epithelial cells, whereas type I IFNs may evoke more systemic antiviral responses.

**Figure 1:**
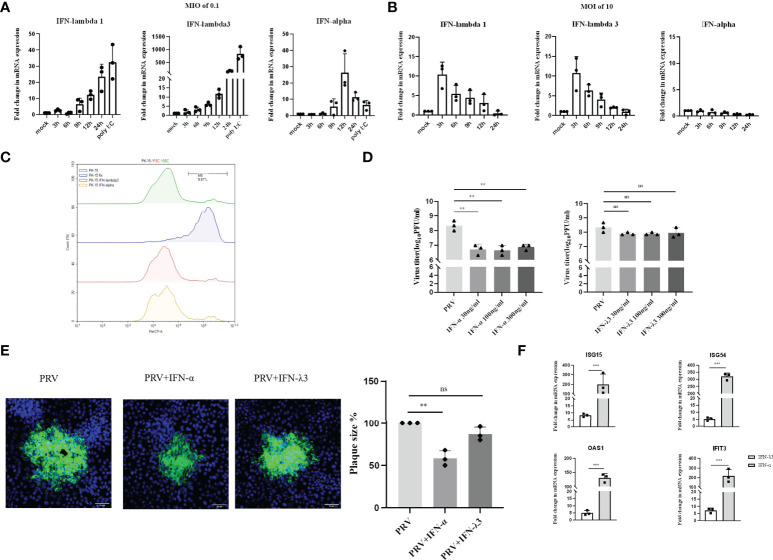
Expression and antiviral effect of type I and type III IFNs in response to PRV infection in PK-15 cells. PK-15 cells were inoculated with **(A)** low dose PRV (MOI of 0.1PFU/cell) or **(B)** high dose PRV (MOI of 10PFU/cell). At different time points post inoculation, cell lysates were prepared for RT-qPCR analysis of expression of porcine IFN-α, IFN-λ1 and IFN-λ3. **(C)** PK-15 cells were treated with IFN-α or IFN-λ3 (300ng/ml) for 24 h, followed by PI staining and flow cytometric analysis to assess cell viability. **(D)** PK-15 cells were pretreated with recombinant porcine IFN-α or IFN-λ3 (30, 100, 300ng/ml) for 24 h prior, followed by PRV inoculation (MOI of 0.1PFU/cell). At 24hpi, cell culture supernatants were collected and viral titers were determined. **(E)** PK-15 cells were pretreated with recombinant porcine IFN-α or IFN-λ3 (300ng/ml) for 24 h, followed by PRV inoculation. At 2hpi, medium was replaced with a semisolid medium to prevent cell-free spread of PRV particles. Plaque sizes were determined by immunofluorescence at 24hpi. **(F)** PK-15 cells were treated with recombinant porcine IFN-α (grey bars) or IFN-λ3 (black bars)(each at 300ng/ml) for 24 h followed by RT-qPCR analysis of the expression of different ISGs (ISG15, ISG54, OAS, IFIT3). Asterisks indicate statistically significant differences (ns: non-significant, **p < 0.01, ***p < 0.001).

PK-15 cells are porcine kidney epithelial cells that are widely used in PRV research. To investigate whether PRV infection induces type I and/or III IFN production in PK-15 cells, mRNA levels of type I IFN (IFN-α) and type III IFN (IFNλ1 and IFN-λ3) were examined by RT-qPCR at 3, 6, 9, 12 and 24hpi at an MOI of 0.1PFU/cell. Substantial levels of IFN-λ1/3 and IFN-α were only detected from 6hpi-9hpi onwards (**Figure 1A**). Interestingly, IFN-α expression reached its peak at 12h and decreased afterwards, whereas expression of both IFNλ1 and IFN-λ3 continued to increase until the end of the experiment at 24hpi (**Figure 1A**). To assess whether expression of type I and/or type III IFN in response to PRV is MOI-dependent, additional assays were performed using an MOI of 10PFU/cell. Inoculation of PK-15 cells at an MOI of 10PFU/cell resulted in a rapid and temporal expression of IFNλ1 and IFN-λ3 (**Figure 1B**). Interestingly, this high MOI inoculation dose did not trigger detectable expression of IFN-α (**Figure 1B**).

Next, we evaluated the antiviral activity of type I and III IFN against PRV in PK-15 cells by assessing infectious virus production and plaque formation. To this end, PK-15 cells were pretreated with different concentrations of porcine recombinant IFN-α or IFN-λ3 for 24 h prior to infection. Treatment with either type I or type III IFN did not negatively affect PK-15 cell viability (**Figure 1C**). To determine infectious virus production, cell culture supernatants were collected at 24hpi and viral titers were determined. Plaque sizes were determined by immunofluorescence at 24hpi. As shown in **Figure 1D**, we observed that PRV titers were significantly reduced upon treatment with IFN-α, whereas IFN-λ3 only minimally and non-significantly reduced PRV titers. No dose-dependent differences were observed, indicating that the lowest concentration (of IFN-α) used was likely sufficient to trigger maximal signaling and/or intracellular antiviral responses. In line with the results of virus titrations, plaque assays showed that IFN-α significantly inhibited PRV spread in PK-15 cells, whereas IFN-λ3 had only a minimal, non-statistically significant effect on PRV plaque sizes in this cell type (**Figure 1E**).

In line with these results, we found that IFN-α triggered much more robust ISG expression in PK-15 cells compared to IFN-λ3 (**Figure 1F**).

These data indicate that PRV triggers expression of both types of IFN in PK-15 cells infected at low MOI with PRV, but only expression of type III IFN in PK-15 cells infected at high MOI. In addition, only type I IFN triggered robust expression of ISGs and significantly suppressed PRV infectious virus production and plaque formation in this cell type.

### In PoREC cells, PRV infection does not trigger a detectable type I or type III IFN response, but both types of interferon display antiviral activity against PRV

**Figure 2:**
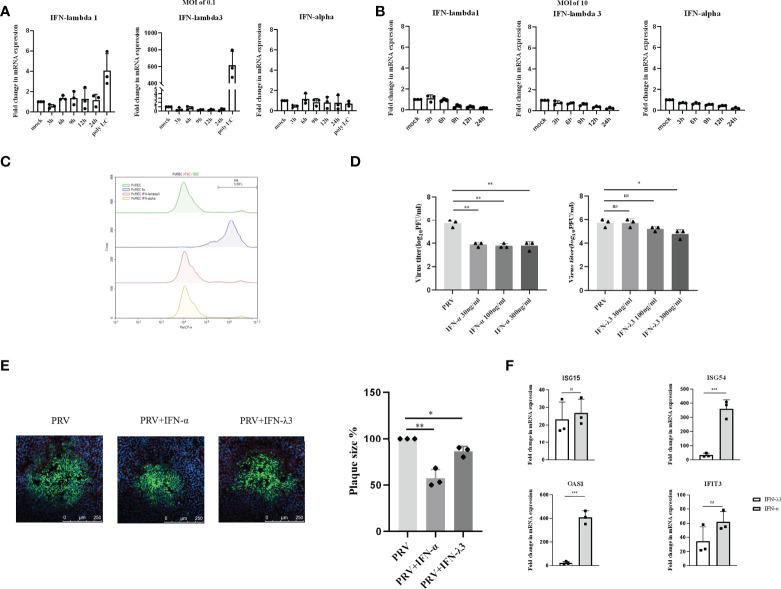
Expression and antiviral effect of type I and type III IFNs in response to PRV infection in porcine primary respiratory epithelial cells (PoREC). PoREC were inoculated with **(A)** low dose PRV (MOI of 0.1PFU/cell) or **(B)** high dose PRV (MOI of 10PFU/cell) on the apical side. At different time points post inoculation, cell lysates were prepared for RT-qPCR analysis of expression of porcine IFN-α, IFN-λ1 and IFN-λ3. **(C)** PoREC were treated with IFN-α or IFN-λ3 (300ng/ml) for 24 h, followed by PI staining and flow cytometric analysis to assess cell viability. **(D)**PoREC were pretreated with recombinant porcine IFN-λ3 or IFN-α (30, 100, 300ng/ml) for 24h on the basolateral side, followed by PRV inoculation on the apical side (MOI of 0.1PFU/cell). At 24hpi, cell culture supernatants were collected and viral titers were determined. **(E)** PoREC were pretreated with recombinant porcine IFN-α or IFN-λ3 (300ng/ml) for 24 h, followed by PRV inoculation. At 2hpi, medium was replaced with a semisolid medium to prevent cell-free spread of PRV particles. Plaque sizes were determined by immunofluorescence at 24hpi. **(F)** PoREC were treated with recombinant porcine IFN-α (grey bars) or IFN-λ3 (black bars)(each at 300ng/ml) for 24 h followed by RT-qPCR analysis of the expression of different ISGs (ISG15, ISG54, OAS, IFIT3). Asterisks indicate statistically significant differences (ns: non-significant, *p < 0.05, **p < 0.01, ***p < 0.001).

Primary respiratory epithelial cells (PoREC) cultured at air-liquid interface (ALI) mimic the natural environment found in respiratory epithelium. This is a particularly relevant model in the context of the early phase of PRV infection, as PRV causes a primary infection in porcine respiratory epithelial cells. We previously showed successful infection of PoREC cells (15). Interestingly, we found that PRV infection at an MOI of 0.1PFU/cell triggers minimal to no detectable mRNA levels of type I or III IFN (**Figure 2A**). Increasing the infectious dose to an MOI of 10PFU/cell still did not trigger detectable mRNA expression of either type of IFN (**Figure 2B**). Quite contrary, PRV infection of PoREC at high MOI appeared to result in mRNA expression levels of type I and type III IFN that were lower than those observed in mock-infected cells (**Figure 2B**).

The expression of type I IFN receptors is largely restricted to the basolateral side of respiratory epithelial cells (23). In addition, other reports used basolateral pretreatment to assess the antiviral activity of type I and type III IFN in respiratory epithelial cells, e.g. against SARS-CoV-2 (24, 25). Hence, to determine whether PRV is sensitive to type I and III IFNs in PoREC, we pretreated the basolateral side of PoREC cultures with IFN-λ3 or IFN-α for 24h, followed by infection of PoREC cultures with PRV from the apical side. Treatment with either type I or type III IFN did not negatively affect PoREC cell viability (**Figure 2C**). Infectious virus production was assessed by collecting cell culture supernatants at 24hpi and determining viral titers. Compared to untreated cells, we observed that both types of IFN suppressed PRV infectious virus production in PoREC. However, IFN-α suppressed infectious virus production more effectively and was equally active over the range of different concentrations tested. IFN-λ3, on the other hand, showed a more limited and dose-dependent antiviral effect, with only the highest concentration resulting in a statistically significant reduction in virus titer (**Figure 2D**). In line with these results, plaque assays showed that IFN-α significantly inhibited PRV spread in PoREC cells, whereas IFN-λ3 had a less significant effect on PRV plaque sizes in this cell type (**Figure 2E**).

In line with these results, we found that IFN-α triggered much more robust ISG expression in PoREC compared to IFN-λ3, particularly ISG54 and OAS1(**Figure 2F**), whereas the expression of ISG15 and IFIT3 were equal or non-statistically different upon treatment with either type of IFN, respectively.

Altogether, these results indicate that PRV-infected PoREC cells do not trigger detectable expression of either type I or type III IFN and that both types of IFN, in particular type I IFN, display antiviral activity against PRV in this cell type.

### In IPEC-J2 cells, PRV infection triggers a rapid and temporal expression of type I and type III IFNs, but only type III IFN displays antiviral activity against PRV

**Figure 3:**
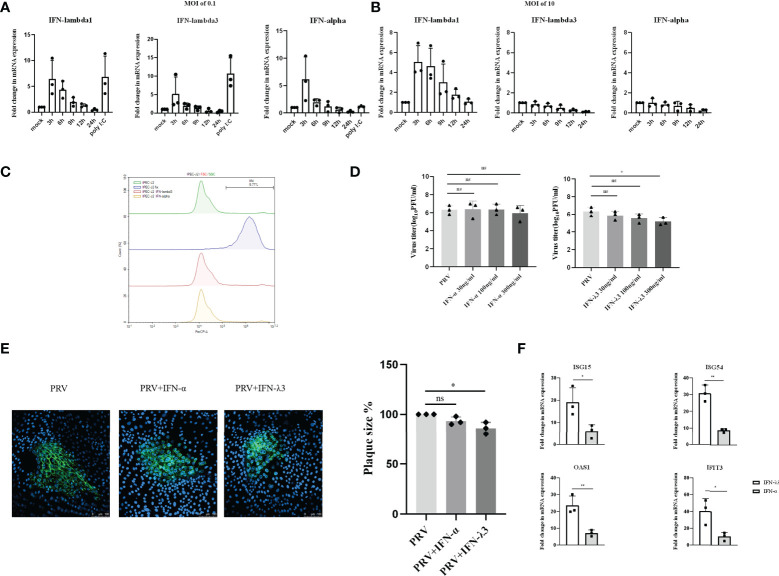
Expression and antiviral effect of type I and type III IFNs in response to PRV infection in IPEC-J2 cells. IPEC-J2 cells were inoculated **(A)** low dose PRV (MOI of 0.1PFU/cell) or **(B)** high dose PRV (MOI of 10PFU/cell). At different time points post inoculation, cell lysates were prepared for RT-qPCR analysis of expression of porcine IFN-α, IFN-λ1 and IFN-λ3. **(C)** IPEC-J2 cells were treated with IFN-α or IFN-λ3 (300ng/ml) for 24 h, followed by PI staining and flow cytometric analysis to assess cell viability. **(D)** IPEC-J2 were pretreated with recombinant porcine IFN-λ3 or IFN-α (30, 100, 300ng/ml) for 24h, followed by PRV inoculation (MOI of 0.1PFU/cell). At 24hpi, cell culture supernatants were collected and viral titers were determined. **(E)** IPEC-J2 cells were pretreated with recombinant porcine IFN-α or IFN-λ3 (300ng/ml) for 24 h, followed by PRV inoculation. At 2hpi, medium was replaced with a semisolid medium to prevent cell-free spread of PRV particles. Plaque sizes were determined by immunofluorescence at 24hpi. **(F)** IPEC-J2 cells were treated with recombinant porcine IFN-α (grey bars) or IFN-λ3 (black bars)(each at 300ng/ml) for 24 h followed by RT-qPCR analysis of the expression of different ISGs (ISG15, ISG54, OAS, IFIT3). Asterisks indicate statistically significant differences (ns: non-significant, *p < 0.05, **p < 0.01).

PRV infection causes diarrhea and necrotizing enteritis in weaning and starter piglets (26, 27). Hence, we next investigated the type I and III IFNs response to PRV infection in porcine intestinal IPEC-J2 cells. We found that, within 3hpi, PRV infection of IPEC-J2 cells at an MOI of 0.1PFU/ml triggers expression of both type I and type III IFN mRNA, followed by a rapid decrease in the corresponding transcript levels (**Figure 3A**). Interestingly, increasing the PRV infectious dose to an MOI of 10PFU/cell resulted in expression of IFN-λ1 but did not result in detectable expression of IFN-α or IFN-λ3 (**Figure 3B**). In fact, PRV infection of IPEC-J2 at high MOI appeared to result in mRNA expression levels of IFN-α or IFN-λ3 that were lower than those observed in mock-infected cells (**Figure 3B**).

To determine whether type I and/or III IFN suppresses PRV infection in IPEC-J2 cells, cells were pretreated with different concentrations of porcine IFN-α or IFN-λ3 for 24h before virus inoculation. Treatment with either type of IFN did not negatively affect IPEC-J2 cell viability (**Figure 3C**). Interestingly, although IFN-α did not suppress PRV infectious virus production in this cell type, IFN-λ3 treatment resulted in a dose-dependent decrease in infectious virus production, which reached statistical significance with the highest dose that was used (**Figure 3D**). In line with this, plaque assays showed that IFN-λ3 significantly inhibited PRV spread in IPEC-J2 cells, while IFN-α did not (**Figure 3E**).

Further in line with these results, we found that IFN-λ3 triggered a much more robust ISG expression in IPEC-J2 cells compared to IFN-α (**Figure 3F**).

In conclusion, in IPEC-J2 cells, PRV infection triggers a MOI-dependent, rapid but temporal expression of both type I and III IFNs, but only type III IFN suppresses PRV infection in this cell type.

### Expression of type I and III IFNs receptor chains

**Figure 4:**
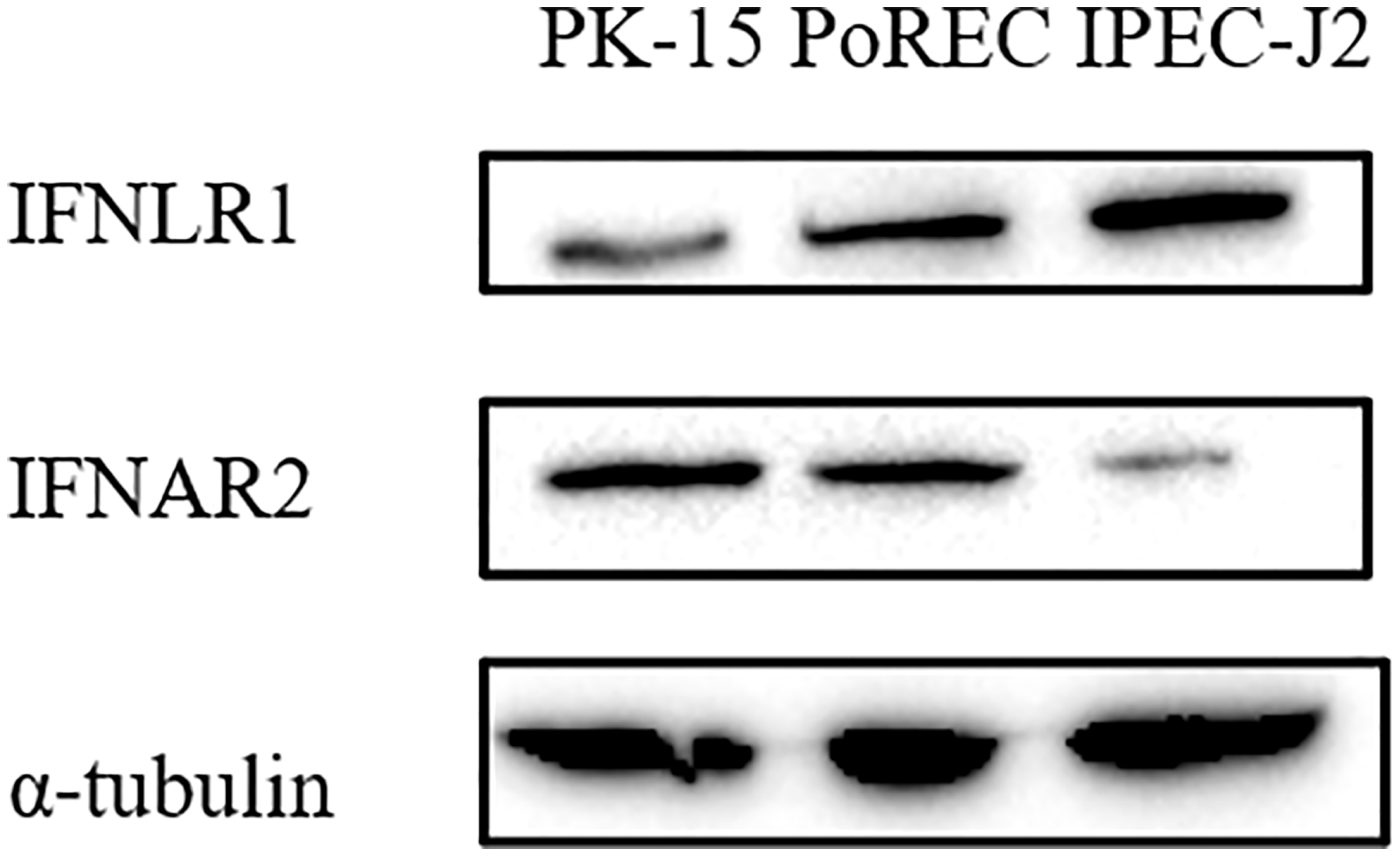
Different porcine epithelial cells show differences in protein expression levels of the type I IFN receptor chain IFNAR2 and type III IFN receptor chain IFNL1. PK-15, PoREC, IPEC-J2 cells were harvested and prepared for Western blot analysis using antibodies against IFNLR1, IFNAR2 and α-tubulin.

Type I and III IFN bind to different receptors, in both cases triggering JAK-STAT signaling pathway to induce ISG expression that mediate antiviral activity. Unlike type I IFN receptors, which are ubiquitously expressed on all nucleated cells, expression of the specific type III IFN receptor chain (IFNLR1) is limited and largely restricted to epithelial cells (6, 28). Since we found that ISG expression and the antiviral effect of IFN-α or IFN-λ3 against PRV depended on the type of epithelial cell, we determined whether this correlated with differences in IFN receptor expression. Expression of the type I IFN receptor chain IFNAR2 and the type III IFN receptor chain IFNLR1 was detected by Western blot in PK-15, PoREC and IPEC-J2 cells. As shown in **Figure 4**, IFNAR2 expression was abundant in PK-15 cells and PoREC but rather weakly expressed in IPEC-J2 cells. IFNLR1, on the other hand, was strongly expressed in IPEC-J2 cells and, to a lesser extent, in PoREC, whereas expression of this receptor chain was relatively weak in PK-15 cells, all in line with the ISG expression and anti-PRV activity of type I and type III IFNs in the different cell types.

## Discussion

Type I and III IFNs are critical antiviral cytokines that are produced as part of the innate immune response to viral infection and are able to establish an antiviral state in host cells. Type III IFN are increasingly regarded to play a unique role in protecting local mucosal surfaces against viral infections, compared to type I IFN which triggers more systemic antiviral responses (29). In this study, we investigated both the expression of type I and III IFN in response to PRV infection and their antiviral effect against PRV infection in different porcine epithelial cells: kidney epithelial cells (PK-15), primary respiratory epithelial cells (PoREC) and intestinal epithelial cells (IPEC-J2). We observed that type I and III IFNs were produced in response to PRV infection in PK-15 cells and IPEC-J2 cells, but that PoREC did not express detectable levels of either type of IFN. A limitation of the current study is that type I and type III IFN mRNA expression levels, rather than protein levels, were analyzed. However, the lack of reliable porcine type III IFN ELISA precluded indisputable detection of porcine type III IFN protein levels. Pretreatment of the different cell types with recombinant porcine IFN-λ3 or IFN-α showed that IFN-α, but not IFN-λ3 inhibited PRV replication in PK-15 cells, whereas the opposite was true for IPEC-J2 cells and both IFN-λ3 and IFN-α suppressed PRV infection in PoREC (although the antiviral effect of IFN-α was more potent in this cell type). These data highlight that, even within the epithelial cell type compartment, substantial differences can be observed both in expression and antiviral activity of type I versus type III IFNs against a viral pathogen like PRV.

PK-15 cells arguably represent the most widely used cell system in PRV studies, as it is very commonly used for virus isolation, propagation and basic research. We found that both type I and type III IFN were induced in response to low MOI PRV infection in PK-15 cells. Interestingly, upon high MOI infection, PRV infection still triggered temporal type III IFN production in PK-15 cells, but did not trigger detectable IFN-α production. Like several alphaherpesviruses, PRV infection of host cells has been reported to rapidly interfere with production of type I IFN. This rapid shutdown of interferon production is carried out by viral tegument proteins that are released during entry of the virus and by (immediate) early viral proteins. For example, the UL13 tegument protein kinase of PRV suppresses type I IFN production *via* degradation of PRDX1, IRF3 and STING (30–32), the US3 tegument protein kinase of PRV triggers degradation of IRF3 and Bclaf1 to suppress type I IFN production (33, 34), and the early EP0 protein of PRV antagonizes type I IFN production by inhibiting IRF9 (35). Hence, although speculative, we hypothesize that, in comparison with low MOI infection, high MOI PRV infection leads to increased intracellular concentration of incoming tegument viral proteins and increased production of (immediate) early viral proteins, which counteract the type I IFN response. Based on our results in PK-15 cells and IPEC-J2 cells, this high MOI-dependent inhibition of IFN production appears to mainly affect type I IFN rather than type III IFN.

We observed that PK-15 cells produce both IFN-λ1 and IFN-λ3 in response to PRV infection, which is in line with observations in PK-15 cells infected with other porcine viruses, including classical swine fever virus (CSFV) and Seneca virus A (SVA) (36, 37). Since porcine type III IFN has been reported to induce antiviral activity against porcine epidemic diarrhea virus (PEDV) infection in Vero E6 cells, which are monkey kidney epithelial cells, and in MARC-145, which are African green monkey kidney cells (19, 38), we assumed that porcine type III IFN may suppress PRV infection in porcine kidney epithelial cells like PK-15 cells. However, although IFN-α significantly inhibited PRV infection, IFN-λ3 did not significantly inhibit PRV infection in PK-15 cells, which corresponded with a very weak induction of ISG expression by IFN-λ3 compared to IFN-α in this cell type. Although the specific receptor chain for type III IFN, IFNLR1, is expressed in PK-15 cells (16), we found that protein expression levels of this receptor chain are less abundant in this cell type compared to PoREC and IPEC-J2 cells (**Figure 4**), in line with the apparent weak ISG induction and antiviral activity of type III IFN in PK-15 cells. Interestingly, very recently, another study reported that porcine IFN-λ3 does display antiviral activity against PRV in PK-15 cells (12). A very important difference of the latter study compared to the current study is that we made use of wild type, virulent PRV whereas the other study made use of an attenuated gEnullgInull PRV strain (12). Since the gE/gI virulence complex of PRV has been reported to interfere with the antiviral IFN system (39, 40), it is likely that the lack of gE and/or gI renders the virus more susceptible to the (relatively weak) antiviral response of IFN-λ3 in PK-15 cells, which may explain the differences between both studies. In addition, an earlier study reported that another type III IFN, IFN-λ1, displays antiviral activity against the wild type PRV strain Ea in PK-15 cells (13). This suggests that, in PK-15 cells, porcine IFN-λ1 may possibly trigger a more robust antiviral effect against PRV compared to IFN-λ3.

The (upper) respiratory tract is a common target for virus entry into the host, including for PRV. In the current study, we isolated porcine primary respiratory epithelial cells (PoREC) from the porcine trachea region. PoREC cultivated at an air-liquid interface have been shown earlier to be permissive to PRV infection (15). We observed a lack of detectable expression of either type I or type III IFN in PoREC following PRV infection, either at low or high MOI infection. Similarly, previous research showed that SARS-CoV-2 does not induce detectable type I and III IFN production or signaling in primary human bronchial/tracheal epithelial cells and human lung alveolar epithelial cells (24, 41, 42), although, using the same primary human bronchial/tracheal epithelial cells system, influenza virus induced a robust type I and III IFNs response (24). Also, human nasal epithelial cells can express both type I and IFNs in response to SARS-CoV-2 infection (43). Meanwhile, porcine primary tracheal epithelial cells did induce both type I and III IFN expression in response to swine IAV infection (44). Hence, the lack of IFN expression in primary porcine trachea epithelial cells following PRV infection appears to be specific for this virus and likely points to a powerful IFN production evasion strategy in this cell type, which warrants further investigation.

Although PoREC cultures did not detectably produce IFN in response to PRV infection, exogenous IFN-λ3 and IFN-α were able to restrict PRV replication in PoREC, and IFN-α provided a more potent antiviral protection against PRV than IFN-λ3 in this cell type. Although speculative, these results may possibly relate to different requirement for type I versus III IFNs in the upper and lower respiratory tract, respectively. Indeed, it has been shown before that type III IFNs appear to be more important than type I IFN to control viral infections at the level of the upper respiratory tract, whereas this may not be the case in the lower respiratory tract. Indeed, using an upper respiratory tract IAV infection model in mice, IFNLR1-/- mice were unable to control IAV spread from the upper airways to the lungs, whereas IFNAR1-/- mice were able to do so, whereas both type I and type III IFNs displayed redundant functions in the lower airway epithelium (45). In the current study, we pretreated PoREC with IFN *via* the basolateral side. Interestingly, aerosol inhalation (which leads to apical exposure) has been assessed as a route of IFN-α2b administration in the context of SARS-CoV-2 infection, and was found to significantly shorten the duration of SARS-CoV-2 shedding (46). Hence, in future assays, it may be of interest to investigate whether apical addition of type III IFN may or may not trigger an antiviral effect of type III IFN against PRV in PoREC.

Upon primary infection at the respiratory tract, PRV may spread to other organs, including the intestinal tract. PRV infection causes diarrhea and necrotizing enteritis in weaning and starter piglets, and PRV antigen can be detected in the intestinal tract (47). The IPEC-J2 cell line is derived from porcine intestinal columnar epithelial cells that were isolated from the mid-jejunum of a neonatal piglet. This cell line displays a high similarity to primary porcine intestinal epithelial cells and is therefore often used as a model to investigate the interaction between viruses and the intestinal tract (48). Our data show rapid and temporal expression of both type I and III IFNs in response to low MOI PRV infection in IPEC-J2 cells. Similarly, PEDV has also been reported to trigger a rapid and temporal expression of type III IFNs in porcine epithelial cells, with detectable expression at 3hpi that quickly declined by 9hpi and 12hpi (19). In human intestinal epithelial cells, type III IFNs were strongly upregulated in response to enteric virus infection, while the expression of type I IFNs was less prominent (49, 50). In line with our observation in PK-15 cells, no IFN-α (or IFN-λ3) production was detected upon high MOI infection of IPEC-J2 cells with PRV, again possibly pointing to the increased activity of IFN production-inhibiting viral tegument and (immediate) early proteins upon high MOI infection.

Interestingly, whereas exogenously added IFN-λ3 significantly restricted PRV replication in IPEC-J2 cells, IFN-α did not, suggesting that type III IFN plays a key role in the antiviral response against PRV in the intestinal tract. In line with this, in IPEC-J2 cells, IFN-λ3 triggered a much more robust expression of ISGs compared to IFN-α and protein levels of the type III IFN receptor chain IFNLR1 were high in IPEC-J2 whereas those of the type I IFN receptor chain IFNAR2 were low. Further in line with these results, using the same IPEC-J2 cells, type III IFN triggered a stronger anti-PEDV effect than type I IFNs, which also correlated with similar differences in ISG expression (21, 38, 51). Exogenous addition of either type of IFN efficiently inhibited SARS-CoV-2 replication and reduced release of infectious virus particles in T84 and Caco-2 human intestinal cell lines. However, depletion of the type I IFN receptor from human intestinal cells resulted in only a slight increase in SARS-CoV-2 infection, whereas depletion of the type III IFN receptor resulted in significantly increased virus infection, replication, and *de novo* virus production (52), again in line with our current results that type III IFN appears to play a critical role in the intestinal antiviral response.

Overall, these data highlight that in specific types of epithelial cells, particularly intestinal epithelial cells, type III IFN provides effective antiviral activity against PRV infection and highlight that the expression as well as the antiviral activity of type I and type III IFNs is highly dependent of the cell type, even within the epithelial cell compartment, and that expression of type I/III IFNs upon PRV infection also depends on the infectious dose that is used.

## Data availability statement

The raw data supporting the conclusions of this article will be made available by the authors, without undue reservation.

## Author contributions

YY and HF conceived and designed the study, YY performed the experiments with support of JM, CVW and BD, YY and HF wrote the paper. All authors contributed to the article and approved the submitted version.

## Funding

YY and JM are supported by PhD scholarship grants of the China Scholarship Council (CSC) (201806910083 and 201906350196). Research of YY and HF is supported by grants from the Special Research Fund of Ghent University (CSC-cofunding grant and Concerted Research Action GOA013-17).

## Acknowledgments

We thank Sofie Denaeghel for help with flow cytometry and Thomas Mettenleiter (Friedrich-Loeffler-Institute, Germany) for providing us with the PRV Kaplan strain.

## Conflict of interest

The authors declare that the research was conducted in the absence of any commercial or financial relationships that could be construed as a potential conflict of interest.

## Publisher’s note

All claims expressed in this article are solely those of the authors and do not necessarily represent those of their affiliated organizations, or those of the publisher, the editors and the reviewers. Any product that may be evaluated in this article, or claim that may be made by its manufacturer, is not guaranteed or endorsed by the publisher.
